# Adsorption Removal of Multiple Dyes Using Biogenic Selenium Nanoparticles from an *Escherichia coli* Strain Overexpressed Selenite Reductase CsrF

**DOI:** 10.3390/nano8040234

**Published:** 2018-04-12

**Authors:** Xian Xia, Zijie Zhou, Shijuan Wu, Dan Wang, Shixue Zheng, Gejiao Wang

**Affiliations:** State Key Laboratory of Agricultural Microbiology, College of Life Sciences and Technology, Huazhong Agricultural University, Wuhan 430070, China; xiaxian@webmail.hzau.edu.cn (X.X.); zijiezhou@webmail.hzau.edu.cn (Z.Z.); shijuanwu@webmail.hzau.edu.cn (S.W.); wangdan_22222@aliyun.com (D.W.); zhengsx@mail.hzau.edu.cn (S.Z.)

**Keywords:** CsrF, Bio-SeNPs, anionic dye, cationic dye, adsorption

## Abstract

Selenite reductase CsrF overexpressed *Escherichia coli* was used as a microbial factory to produce Se(0) nanoparticles (Bio-SeNPs). The Bio-SeNPs were characterized by transmission electronic microscopy, element mapping, scanning electron microscopy, energy-dispersive X-ray spectrographs, Zeta-potential, dynamic light scattering, Fourier transform infrared spectroscopy and X-ray photoelectron spectroscopy analyses. The results indicated that Bio-SeNPs are irregular spheres with diameters from 60 to105 nm and mainly consist of Se(0), proteins and lipids. Furthermore, it exhibited maximum adsorption capacity for anionic dye (congo red) at acidic pH and cationic dyes (safranine T and methylene blue) at alkaline pH. To gain more insight, adsorption kinetics, adsorption isotherms and adsorption thermodynamics studies were carried out. These results showed that the adsorption capacities of congo red, safranine T and methylene blue were 1577.7, 1911.0 and 1792.2 mg/g, respectively. These adsorption processes were spontaneous and primarily physical reactions. In addition, Bio-SeNPs can be effectively reused by 200 mmol/L NaCl. To the best of our knowledge, this is the first report of adsorption removal dyes by Bio-SeNPs. The adsorption capacities of Bio-SeNPs for congo red, safranine T and methylene blue were 6.8%, 25.2% and 49.0% higher than that for traditional bio-based materials, respectively.

## 1. Introduction

Dyes are chemical compounds that bind to surfaces or fabrics to impart color [1]. Based on their electric charge, dyes are classified into anionic (acidic) and cationic (basic) moieties. They are widely used in the textile, leather tanning, cosmetic, printing and other manufacturing industries [2]. The extensive use of dyes, however, causes pollution to many bodies of water in the environment [3]. Most dyes exhibit high-intensity colors, which are harmful to some aquatic life because they cause a decrease in light penetration [1]. Moreover, dyes are carcinogenic, mutagenic or teratogenic to various organisms and cause damage to the kidney, reproductive system, liver, brain and central nervous system in human beings [1,4]. Therefore, removal of dyes from water is urgently needed. 

Numerous physical, chemical and biological methods have been used for dye removal [5]. Of these, adsorption is a flexible, simple and low-cost method [6]. Materials such as graphene, polymers with intrinsic microporosity, imprinted polymers and zeolites are used as adsorbents for sustainable wastewater treatment [7]. Moreover, sustainable adsorption separations technology is also applied in the enrichment of rare earth [8], oleuropein [9] and lactic acid recovery [10]. Recently, bio-based materials have been paid increasing attention due to their applications in environmental remediation and other fields [11,12]. In order to take advantages of the low cost and environmentally friendly character of bio-based materials [13], it is necessary to develop novel and efficient bio-techniques. 

Various microorganisms are capable of reducing selenite [Se(IV)] to Se(0) and producing extracellular or intracellular Bio-SeNPs [14]. Enzymatic reduction is a general mechanism in bacteria, and numerous reductases have been reported to catalyze the reduction of selenite, including sulfite reductase [15], nitrite reductase [16], arsenate reductase [17], fumarate reductase [18] and chromate/selenite reductase CsrF [19]. These reductases may also play an important role in the formation and stabilization of Bio-SeNPs [14]. Interestingly, Bio-SeNPs have been applied in various fields as a green nanomaterial, including in anti-cancer activities [20], nutritional supplements [21], anti-microbial applications [22] and drug delivery [23]. In addition, Bio-SeNPs have been reported to be good materials for the adsorption of Zn(II) [24] and Cd(II) [25]. However, to the best of our knowledge, no systematic study of Bio-SeNPs as an absorbent for dye removal has been published, yet. 

Previously, we discovered a selenite and chromate reductase CsrF in *Alishewanella* sp. WH16-1 [19]. In addition, a CsrF overexpression strain *Escherichia coli* S17-1-pCT-Zori-*csrF* was constructed [19]. This strain showed high efficiency in selenite reduction. In this study, we extracted the Bio-SeNPs from the reconstructed *Escherichia coli*. Based on comprehensive analysis, the extracted Bio-SeNPs exhibited a great ability for anionic and cationic dye removal. 

## 2. Materials and Methods 

### 2.1. Materials

The bacterium *Escherichia coli* S17-1-pCT-Zori-*csrF* was constructed as previously described [18]. Methyl orange and methylene blue were provided by Aladdin Shanghai Biochemical Technology (Shanghai, China) Co. Ltd. Congo red, methyl blue and safranine T were purchased from Sinopharm Chemical Reagent (Shanghai, China) Co., Ltd. Azure I and acid red 18 were obtained from Shanghai Macklin Biochemical (Shanghai, China) Co., Ltd. Orange G6 and rose bengal were provided by Shanghai Yuanye Bio-Technology (Shanghai, China) Co., Ltd. 

### 2.2. Bio-SeNPs Production and Extraction

*Escherichia coli* S17-1-pCT-Zori-*csrF* was cultured at 37 °C in Luria-Bertani (LB) medium under aerobic conditions with shaking at 150 rpm. When OD_600_ reached 0.8–1.0, the cells were incubated with Na_2_SeO_3_ (1 mmol/L) and cultured for another 36 h. Next, the cultures were collected by centrifugation (8000 rpm, 10 min) and washed twice with ddH_2_O. Then, the previously described Bio-SeNP extraction process was followed, with a slight modification [19]. One-liter cultures were collected, and the cells were resuspended in ddH_2_O (15 mL). Then, samples were sonicated for 10 min. After 5 min centrifugation at 12,000 rpm, the pellets were washed twice with ddH_2_O and resuspended in ddH_2_O (5 mL). Later, the resuspended pellets were added to 80% sucrose (10 mL). The supernatant was removed after 5 min centrifugation at 12,000 rpm. The pellets were washed and resuspended in ddH_2_O (3 mL). To determine the concentration of the Bio-SeNPs, they were dried overnight in an oven at 80 °C. 

### 2.3. Characterization of Bio-SeNPs

In order to observe the location of Bio-SeNPs in the cells, *Escherichia coli* S17-1-pCT-Zori-*csrF* cells was incubated until the OD_600_ reached 0.8–1.0. After adding Na_2_SeO_3_ for 24 and 36 h, the cells were collected. Then, samples for transmission electronic microscopy (TEM) were prepared, and the measurements were carried out as previously described [19]. Briefly, culture samples were fixed in osmic acid and dehydrated in a series of ethanol (70%, 96% and 100%). The specimens were then embedded in epoxy and cut with a ReichertJung Ultracut E microtome. TEM observation was performed after staining with uranyl acetate and lead citrate.

To further characterize the Bio-SeNPs, the Bio-SeNPs were extracted as described in Section 2.2. Next, TEM, element mapping, scanning electron microscopy (SEM), energy-dispersive X-ray spectrographs (EDS), Zeta-potential, dynamic light scattering (DLS), Fourier transform infrared spectroscopy (FT-IR) and X-ray photoelectron spectroscopy (XPS) analyses were conducted. The high-angle annular dark field (HAADF) scanning TEM (STEM) images and the elemental mapping of C, N, O, P, S and Se were obtained on a Tecnai G2 F20 S-TWIN microscope (FEI Company, Hillsboro, OR, USA). Zeta-potential and DLS were analyzed using a Zen 3600 Zetasizer Nano ZS from Malvern Instruments (Worcestershire, UK). For SEM-EDS, FT-IR and XPS analyses, Bio-SeNPs were dried with a SJIA-10N vacuum freeze dryer (Ningbo YinZhou Sjia Lab Equipment Co., Ltd., YinZhou, China). SEM and EDS were obtained from a JEOL Model JSM-6390LV (Tokyo, Japan). FT-IR and XPS analyses were carried out as previously described [19].

### 2.4. Adsorption Experiments

The experiments were performed in glass bottles on a shaker at 200 rpm. To obtain the optimum pH for adsorption, Bio-SeNPs (0.4 g/L) were incubated with the dyes (200 mg/L; congo red, safranine T and methylene blue) in an initial pH range of 5–10. The initial pH of the dye solutions was adjusted by a 0.1 mmol/L HCl or NaOH solution. Samples were harvested by centrifugation (12,000 rpm for 5 min) after 1 h of adsorption. The amount of congo red, safranine T and methylene blue were detected at 497 nm [26], 520 nm [27] and 660 nm [28], respectively, by a Synergy HT microplate reader (Bio-Tek, Winooski, VT, USA). The adsorbed amount of dye was calculated according to the following Equation (1),

*q_t_* = (*C*_0_ − *C_t_*)*V*/*m* (1)

where *q_t_* is the adsorbed amount after time *t*, *C*_0_ and *C_t_* are the initial and residual concentrations after time *t*, respectively, *V* is the volume of the solution, and *m* is the weight of the Bio-SeNPs used.

To obtain the adsorption capability, Bio-SeNPs (0.4 g/L) were incubated with congo red, safranine T and methylene blue (50 mg/L) at 313 K and the optimized pH for 1 h. The remaining amount of dye was measured at wavelengths of 400–800 nm by a UV spectrophotometer (DU 800, Beckman, Indianapolis, IN, USA).

For adsorption kinetics study, Bio-SeNPs (0.4 g/L) was cultured with various concentrations (50, 100 and 200 mg/L) of congo red, safranine T and methylene blue dyes at 313 K and optimum pH for 1 h, respectively. Samples were collected at the designed time. The remaining amount of dyes was measured as described above.

For the study of adsorption isotherms, Bio-SeNPs (0.2 g/L) was cultured with a series of concentrations of congo red, safranine T and methylene blue dyes at 303, 313 and 323 K at optimum pH for 2 h. The residual dyes were determined by the methods described above. 

### 2.5. Desorption Experiments

For the desorption study, 50 mg/L safranine T and methylene blue were adsorbed on 0.4 g/L Bio-SeNPs under the optimized adsorption conditions. Then, the samples were collected by 8000 rpm, 10 min centrifugation. Various concentration of NaCl (5, 50 and 200 mmol/L) were used to resuspend the samples and regenerate the Bio-SeNPs. The desorption efficient was calculated by the following equation:

Desorption percentage (%) = Concentration desorbed/Concentration adsorbed × 100%(2)

In order to test the reusability of the Bio-SeNPs, five successive adsorption/desorption cycles were performed. The removal efficiency of each cycle was calculated as following:

Removal percentage (%) = Concentration adsorbed/Initial Concentration × 100%(3)

## 3. Results and Discussion

### 3.1. Bio-SeNPs Produced by Escherichia coli S17-1-pCT-Zori-csrF

In our previous study, CsrF was identified as an efficient selenite reductase [19]. The CsrF overexpressed strain *Escherichia coli* S17-1-pCT-Zori-*csrF* had a selenite reduction efficiency that was 20%, 50% and 90% higher that of *Alishewanella* sp. WH16-1 [19], *Escherichia coli* S17-1 wild-type strain [18], and *Stenotrophomonas maltophilia* SeITE02 [29], respectively. The Bio-SeNP synthesis efficiency is positively correlated with selenite reduction efficiency. Generally, the fermentation technology of reconstructed *Escherichia coli* strains is very mature, and it is extensively applied in industrial and agricultural production [30]. Consequently, our strain is a good choice for Bio-SeNPs production. 

Hence, *Escherichia coli* S17-1-pCT-Zori-*csrF* was selected as the microbial factory for the production of Bio-SeNPs in this study. On average, 30 mg of dried Bio-SeNPs were obtained from 1 L of LB broth added to selenite (1 mmol/L). This is a high output compared to *Alishewanella* sp. WH16-1 (about 20 mg Bio-SeNPs per 1 L broth under the same condition). Moreover, the extraction of the Bio-SeNPs is simple. 

**Figure 1:**
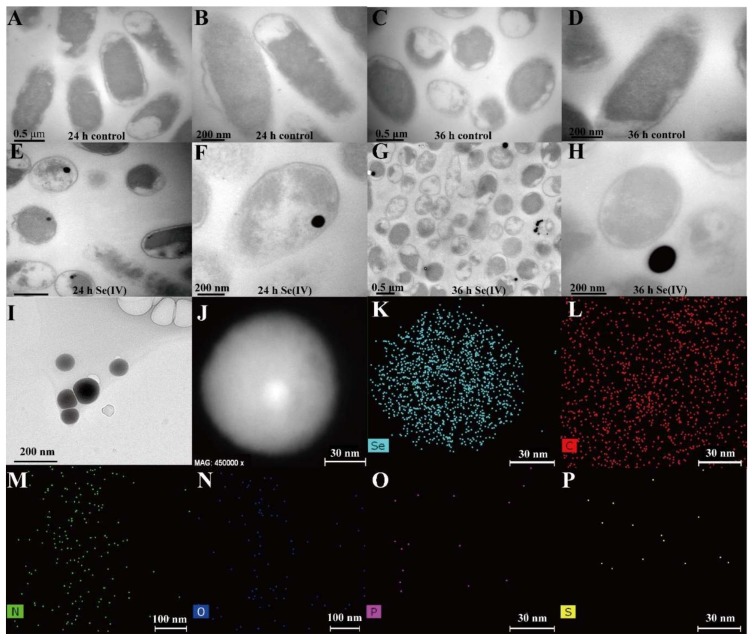
TEM and element mapping analysis of Bio-SeNPs. The *Escherichia coli* S17-1-pCT-Zori-*csrF* strain was cultured until OD_600_ reached 0.8–1.0. Then, the cells were incubated without selenite for 24 h (**A**,**B**) and 36 h (**C**,**D**) and with selenite (1 mmol/L) for 24 h (**E**,**F**) and 36 h (**G**,**H**); The Bio-SeNPs were extracted from the cells (**I**); The HAADF (**J**) and Se, C, N, O, P and S elemental mapping (**K**–**P**) images of Bio-SeNPs were obtained.

TEM was carried out in order to analyze the location of Bio-SeNPs in the cells. The TEM images indicate that at 24 h, most of Bio-SeNPs were located in the intracellular space (Figure 1A,B,E,F). At 36 h, however, the majority of Bio-SeNPs were located in the extracellular space (Figure 1C,D,G,H). This phenomenon is similar to that observed in *Thauera selenatis* [31]. The extracellular deposited Bio-SeNPs may be caused by cell lysis or effluxed by some special secretion systems [31]. 

### 3.2. Characteristics of SeNPs

**Figure 2:**
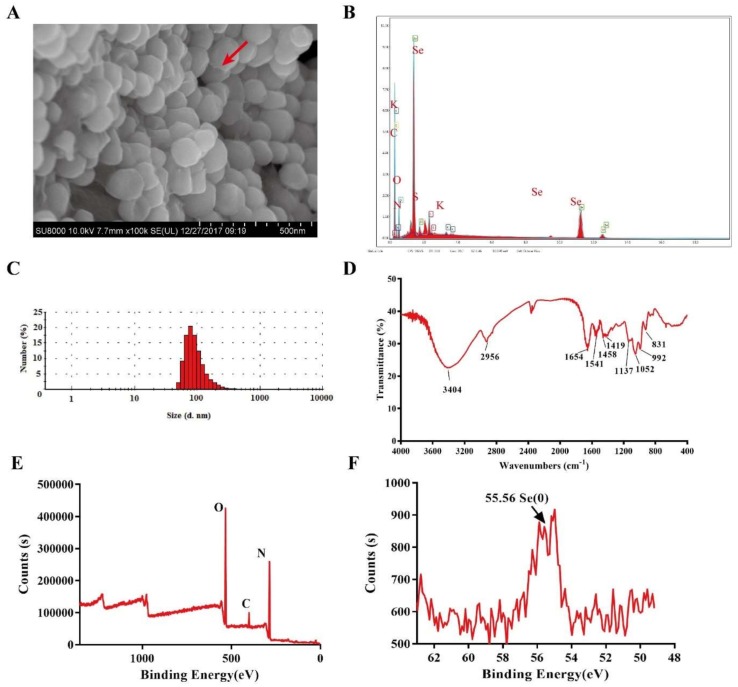
Characterization of Bio-SeNPs. The SEM (**A**) and EDS (**B**) showed that the SeNPs were irregular spheres with a high content of elemental Se. The DLS spectrum (**C**) indicated the diameters of the nanoparticles. The FT-IR (**D**) and XPS (**E**,**F**) spectra exhibited proteins, lipids, inorganic ions and Se(0) in Bio-SeNPs.

TEM (Figure 1I,J), SEM (Figure 2A) and DLS (Figure 2C) showed that the Bio-SeNPs extracted from *Escherichia coli* S17-1-pCT-Zori-*csrF* appeared as irregular spheres with diameters from 60 to 105 nm (77.2%). This size range is similar to *Shewanella oneidensis* MR-1 [18] and smaller than the Bio-SeNPs produced by *Comamonas testosteroni* S44 [32], *Stenotrophomonas maltophilia* SeITE02 [29] and *Alishewanella* sp. WH16-1 [19]. The TEM elemental mapping (Figure 1J–P) and SEM-EDS (Figure 2B) results showed that the Bio-SeNPs contained Se, C, N, O, P and S, some of which may belong to organic substances such as protein and lipids; this result was further supported by the FT-IR analysis (Figure 2D). The FT-IR spectrum showed stretching bands at 3404 (hydroxyl group), 2956, (saturated aliphatic group), 1654 (olefinic alkene group), 1541 (aliphatic nitro compounds), 1458 (aromatic ring stretch), 1419 (organic sulfate), 1052 (primary amine, CN stretch), 992 (aliphatic phosphates) and 831 (aromatic ring group) [33]. In addition, the XPS spectrum exhibited Se3d peaks at 55.56 eV, which was attributed to Se(0) [18]. This indicates that the Se(0) nanoparticles were produced after undergoing selenite reduction by *Escherichia coli* S17-1-pCT-Zori-*csrF*. 

### 3.3. Effects of pH on Zeta Potential for Dye Removal

**Figure 3:**
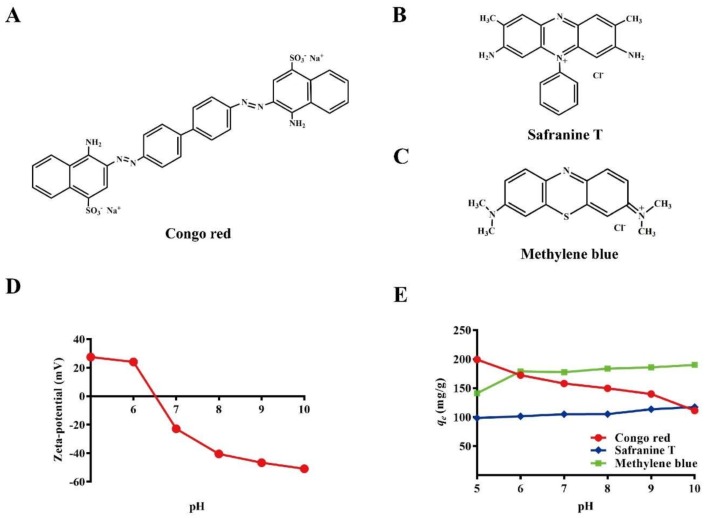
Chemical structure of dyes (**A**) congo red; (**B**) safranine T; and (**C**) methyle blue and the effects of pH on the Zeta potential (**D**) and dye removal capacity (**E**) for Bio-SeNPs. Bars represent the mean ± SD from three independent experiments.

The Zeta potential of Bio-SeNPs at various pH values is shown in Figure 3D. The Zeta potential was positive at low pH (≤6) and negative at high pH (≥7). As pH increased, the Zeta potential decreased. A similar decreasing tendency for Bio-SeNPs has also been identified in previous studies [24,25]. The positive Zeta potential at low pH values may be caused by the protonation of amines. The negative Zeta potential may be caused by the deprotonation of amines, as well as the phosphate/sulfate groups on the surface [26]. 

To analyze the dye removal, numerous anionic and cationic dyes were tested. Bio-SeNPs showed good removal ability for some anionic (congo red and rose bengal) and cationic (methylene blue, safranine T, Azure I) dyes. However, no obvious removal was observed for acid red 18, orange G6 or methyl orange. Congo red (Figure 3A), safranine T (Figure 3B) and methylene blue (Figure 3C) were selected for further detailed study. The removal efficiency of the three selected dyes was examined at various pH values. The results indicate that the removal efficiency of congo red decreased with increasing pH (Figure 3E). In contrast, the removal efficiency of safranine T and methylene blue increased with increasing pH (Figure 3E). This phenomenon was in agreement with the change in Zeta potential at various pH values. At low pH, Bio-SeNPs had a positive potential and showed stronger adsorption of the anionic dye (congo red). At high pH, Bio-SeNPs had a negative potential and exhibited stronger adsorption to cationic dyes (methylene blue and safranine T). This characteristic was consistent with other nanomaterials used for dye removal by adsorption, such as mCS/CNT [34] Fe3O4@GPTMS@Lys [35] and nanocellulose [26]. The optimum pH values for the removal of congo red, safranine T and methylene blue removal were 5, 10, and 10, respectively. The subsequent congo red adsorption study was carried out at pH 6, because the color of the congo red solution changed when pH < 5.5. 

**Figure 4:**
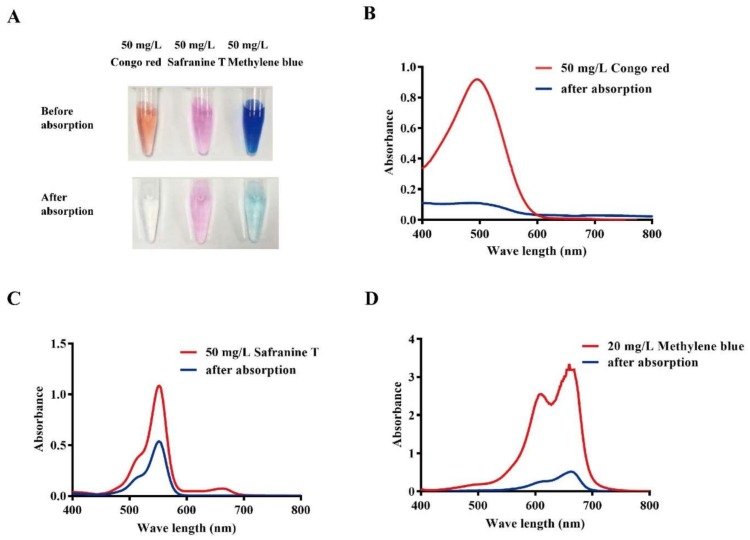
Characterization of dye removal by Bio-SeNPs. Images of the dye solutions after 1 h adsorption (**A**); The blue lines represent the spectra of 50 mg/L of congo red (**B**); safranine T (**C**) and methylene blue (**D**) after removal. The red lines are the spectra of standard solutions of 50 mg/L congo red (**B**); 50 mg/L safanine T (**C**) and 20 mg/L methylene blue (**D**), which were used as controls.

To gain more insight, the solutions were analyzed after adsorption. As shown in Figure 4A, images were captured before/after adsorption and UV spectra of the residual dyes were analyzed. The characteristic peaks of congo red (Figure 4B), safranine T (Figure 4C) and methylene blue (Figure 4D) were markedly decreased after adsorption. The removal efficiencies of methylene blue and congo red were noticeably higher than safranine T.

### 3.4. Adsorption Kinetics

**Figure 5:**
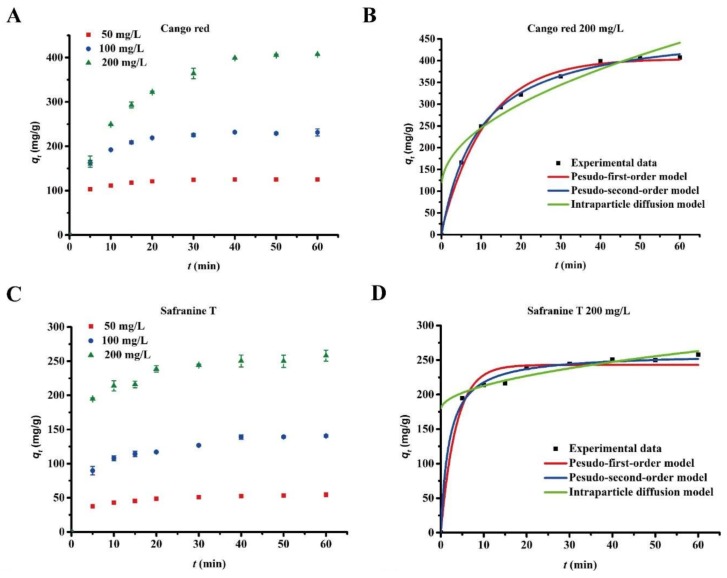

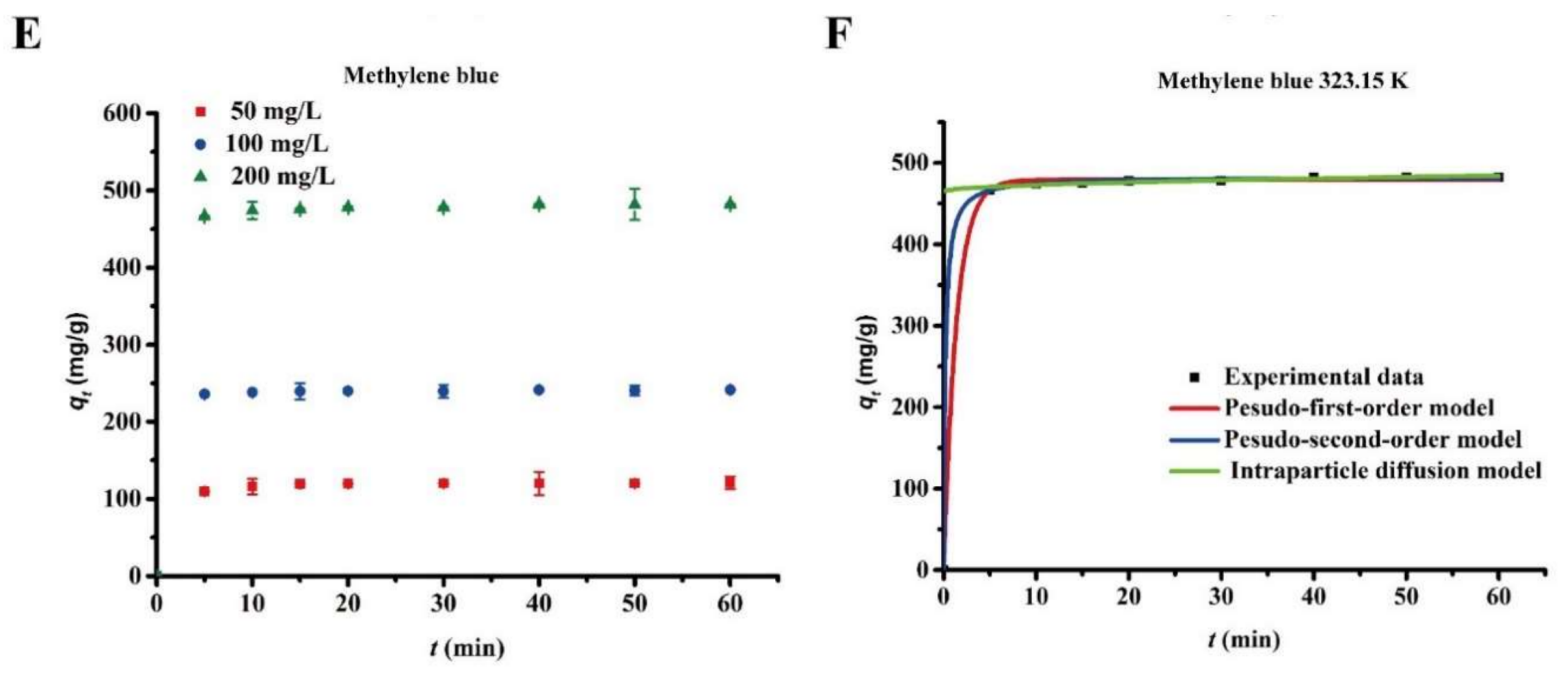
Effect of contact time on the adsorption of dyes at different initial concentrations. 50, 100 and 200 mg/L of congo red (**A**); safranine T (**C**); and methylene blue (**E**) were completely removed by 0.4 g/L of Bio-SeNPs. Data are shown as the mean ± SD of three replicates. The average values of congo red (**B**); safranine T (**D**); and methylene blue (**F**) with initial concentrations of 200 mg/L were used for the pseudo-first-order, pseudo-second-order and intraparticle diffusion model fitting.

To study the adsorption kinetics of Bio-SeNPs for congo red, safranine T and methylene blue, the effect of contact time on the adsorption capacity was tested using three dye concentrations. Based on the results above, the experimental initial pH values were selected at the preferred values of 6, 10 and 10 for congo red, safranine T and methylene blue, respectively. The results showed fast adsorption rates for congo red (Figure 5A), safranine T (Figure 5B) and methylene blue (Figure 5C). In particular, methylene blue only required 5 min of contact time for total adsorption at various initial concentrations (50, 100 and 200 mg/L). Congo red and safranine T took 35 and 25 min more, respectively, to reach equilibrium at a concentration of 200 mg/L than at a concentration of 50 mg/L. This suggests that the adsorption of congo red and safranine T is dependent on the initial concentration. 

Furthermore, pseudo-first-order, pseudo-second-order and intraparticle diffusion models were performed to elucidate the adsorption kinetics. The three models were calculated as follows [35]:

Pseudo-first-order kinetic model: log(*q_e_* − *q_t_*) = log*q_e_* − *k*_1_*t*/2.303 (4)

Pseudo-second-order kinetic model: *t*/*q_t_* = 1/(*k*_2_*q_e_*^2^) + *t*/*q_e_* (5)

Intraparticle diffusion model: *q_t_* = *k*_3_*t*^0.5^ + *c* (6)

where *q_e_* and *q_t_* are the amount of adsorbed dye per unit mass of adsorbent (mg g^−1^) at equilibrium and time *t*, respectively. *k*_1_, *k*_2_ and *k*_3_ are the rate constants of the pseudo-first-order adsorption (h^−1^), pseudo-second-order adsorption (g mg^−1^ h^−1^) and intraparticle diffusion (mg g^−1^ h^−0.5^), respectively. *c* indicates the thickness of the boundary layer. 

The kinetic parameters are shown in Table S1, and the fitting curves at an initial concentration for 200 mg/L are shown in Figure 5D,E,F. The *R*^2^ of the pseudo-second-order equation ranged from 0.9984 to 0.9991, 0.9584 to 0.9962 and 0.9996 to 0.9999 for congo red, safranine T and methylene blue (Table S1), respectively. These *R*^2^ values were higher than those obtained by the pseudo-first-order and intraparticle diffusion models. Moreover, the equilibrium adsorption values calculated by the second-order model were similar to the experimental adsorption results. This finding suggested that the adsorption kinetics of Bio-SeNPs for dyes (congo red, safranine T and methylene blue) fit the pseudo-second-order model. The pseudo-second-order model includes three stages in the adsorption process: membrane diffusion, surface adsorption and internal diffusion. This model better represents this particular adsorption process [36], indicating that the absorption of dyes by Bio-SeNPs is a complex process. Similar adsorption characteristics have been observed for magnetically retrievable chitosan/carbon nanotubes [34] and modified activated carbon [36]. 

### 3.5. Adsorption Isotherms

It is important to understand the equilibrium adsorption isotherm in order to fully understand the adsorption properties of Bio-SeNPs. The experimental data for the Bio-SeNPs were applied to Langmuir, Freundlich and Temkin isotherm equations [37].

Langmuir equation: *C_e_*/*q_e_* = *C_e_*/*q_m_* + 1/*K_L_q_m_* (7)

Freundlich equation: *q_e_* = *K_F_C_e_*^1/^*^n^* (8)

Temkin equation: *q_e_* = *A* + *B*ln*C_e_* (9)

where *q_e_* (mg/g) is the equilibrium adsorption capacity of dye on the Bio-SeNPs, *Ce* (mg/L) is the equilibrium dye concentration in solution, *q_m_* (mg/g) is the maximum capacity of the adsorbent, *K_L_* (L/mg) is the Langmuir adsorption equilibrium constant related to the energy of adsorption, *K_F_* (L/mg) and 1/*n* are the Freundlich constant representing the adsorption capacity and adsorption intensity and *A* and *B* are Temkin constants. 

**Figure 6:**
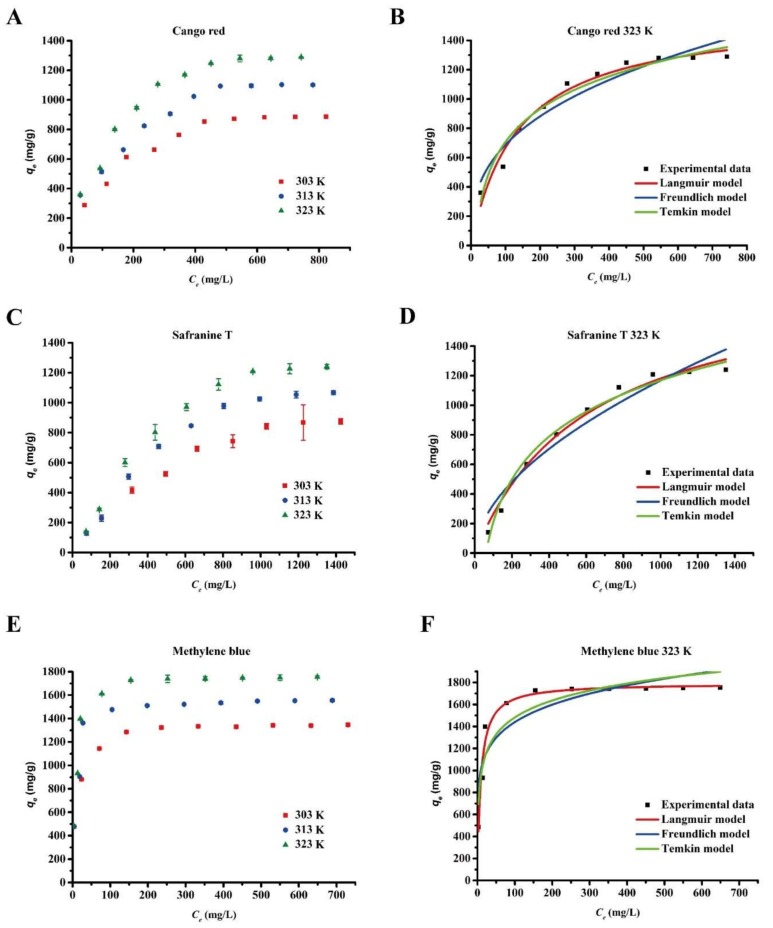
Equilibrium isotherms of the dyes after adsorption by Bio-SeNPs. Various concentrations of congo red (**A**); safranine T (**C**) and methylene blue (**E**) were removed by 0.2 g/L Bio-SeNPs at 303, 313 and 323 K, respectively. Data are presented as mean ± SD of three replicates. Langmuir, Freundlich and Temkin models of congo red (**B**); safranine T (**D**) and methylene blue (**F**) adsorption onto Bio-SeNPs were fitted using average values at 323 K.

Experimental adsorption data were obtained at three different temperatures (303 K, 313 K, and 323 K) (Figure 6A–C). The adsorption isotherm fitting parameters are listed in Table S2 and the fitting curve at 323 K is illustrated in Figure 6D–F. According to the correlation coefficients (*R*^2^, Table S2), the adsorption behavior is consistent with the Langmuir model over the Freundlich and Temkin models. According to the Langmuir model, the maximum quantities (*q_m_*) of congo red, safranine T and methylene blue are 1577.7, 1911.0 and 1792.2 mg/g, respectively. A Freundlich constant of *n* > 2 indicates an easy adsorption process [36]. The value of n for safranine T was lower than 2, while the *n* values for methylene blue and congo red were higher than 2. This finding suggests that Bio-SeNPs have higher adsorptivity for congo red and methylene blue than for safranine T. To reach an identical adsorption quantity, a greater initial concentration of safranine T was required (Figure 6A–C). 

### 3.6. Adsorption Thermodynamics

Usually, thermodynamic analysis provides insight into the spontaneity, randomness, endothermicity or exothermicity of the adsorption process. Based on the Langmuir adsorption equilibrium constant, *K_L_*, the thermodynamic parameters can be calculated according to the van’t Hoff equation:

ln*K_L_* = −Δ*H*^θ^/*RT* + Δ*S*^θ^/*R* (10)

where Δ*H*^θ^ is the enthalpy change (J/mol), Δ*S*^θ^ is the entropy change (J/mol), *R* is the universal gas constant (8.314 J/mol K), *T* is the temperature (K) and *K_L_* (L/mol) is the equilibrium constant obtained from Langmuir model. The values of Δ*H*^θ^ and Δ*S*^θ^ are determined from the slope and intercept of the linear plot of ln*K_L_* vs 1/*T* (inverse temperature), respectively. The obtained Δ*H*^θ^ and Δ*S*^θ^ can then be used to calculate the Gibbs Free Energy, Δ*G*^θ^ (kJ/mol), using the following equation:

Δ*G*^θ^ = Δ*H*^θ^ − *T*Δ*S*^θ^ (11)

The values of the thermodynamic parameters are listed in Table 1. The values of Δ*G*^θ^ were negative at various temperatures. This indicates that the adsorption of the dyes onto Bio-SeNPs is a spontaneous process. The decrease of Δ*G*^θ^ as the temperature increased suggests that the dye adsorption process is more favorable at higher temperatures. The Δ*H*^θ^ of congo red, safranine T and methylene blue were 1.4546, 6.2332 and 8.5850 kJ/mol, respectively. These values were all positive, which means that the adsorption reactions were endothermic. The values of Δ*H*^θ^ were in the range of 1–9 kJ/mol. The variation in enthalpy is primarily caused by physical adsorption [38], which may be caused by electrostatic or Van-der-Waals forces [39]. In addition, all the Δ*S*^θ^ values were positive, which suggested that the randomness at the solid-solution interface increased during the adsorption process. Although the loss of the dye molecules may lead to a decrease in entropy, absorbed solvent molecules could increase the entropy [34]. Furthermore, the increase in entropy may be caused by the electrostatic interaction between the adsorbent and adsorbate, according to previous reports [34,40].

### 3.7. Desorption and Reusability Study

**Figure 7:**
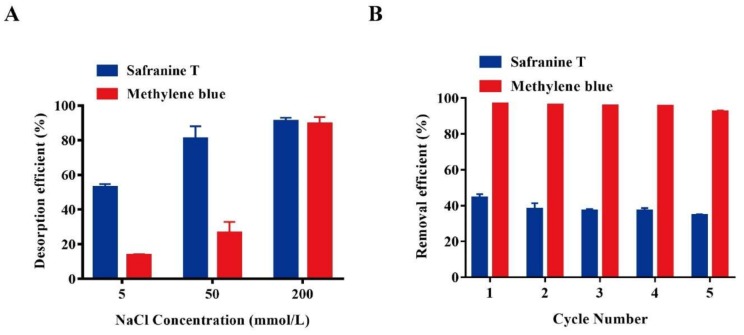
Effects of NaCl concentrations on desorption (**A**) and reusability adsorption (**B**) of Bio-SeNPs. Data are presented as mean ± SD of three replicates.

Desorption and reuse of adsorbents are very important in practical application [41]. Hence, safranine T and methylene blue were selected to study the reusability of Bio-SeNPs. Proteins are vital constituents of Bio-SeNPs, according to the element mapping and FT-IR results in this study and in another recent study [42]. HCl, NaOH and alcohol may inactivate proteins and damage the structure and function of Bio-SeNPs. Therefore, NaCl was used to desorb dyes from Bio-SeNPs. The desorption efficiency reached about 90% at 200 mmol/L NaCl. NaCl in the concentration range of 5–200 mmol/L, as exhibited by the desorption capacity (Figure 7A). The desorption phenomenon was similar to the previously reported cellulose nanofibril aerogels [43]. To assess the reusability, consecutive adsorption/desorption cycles were performed. After five cycles, the removal efficiency of Bio-SeNPs for safranine T and methylene blue were decreased only by about 10% and 5%, respectively (Figure 7B). 

### 3.8. Comparison with Other Adsorbents

A comparison of the adsorption capacity of Bio-SeNPs and other bio-based adsorbents is summarized in Table 2. The adsorption capacities of Bio-SeNPs for congo red, safranine T and methylene blue were 6.8%, 25.2% and 49.0% higher than those for traditional bio-based materials, respectively. Generally, acid hydrolysis [26] and high-temperature (700–800 °C) processing [44] were used to activate the bio-based material. These processes may cause environmental problems or lead to high energy consumption. Meanwhile, fermentation production of Bio-SeNPs is a low energy consumption process. However, the Bio-SeNPs showed no obvious adsorption of acid red 18, orange G6 and methyl orange according to our results. Some other bio-based materials have good adsorption capacity for these dyes [1]. Consequently, different bio-based materials can be combined to deal with pollution from multiple dyes in practical application. 

In this study, the potential dye-removal ability of Bio-SeNPs has been exhibited at the laboratory scale. Based on the results, the industrial application of Bio-SeNPs is promising in the future. Bio-SeNPs can be produced by microbial fermentation of bacterial strain and separated by from fermentation broth by high-pressure homogenization and centrifugation. The adsorption ability of Bio-SeNPs is stable when stored at 4 °C for at least 4 weeks. Bio-SeNPs can be fixed in equipment with columns [7] for dye removal. Special membranes can be used for nanometer-size separation [45]. 

## 4. Conclusions and Perspective

In this study, the CsrF overexpression strain *Escherichia coli* S17-1-pCT-Zori-*csrF* showed high Se(IV) reduction ability, which ultimately produced Bio-SeNPs. These nanoparticles are irregular spheres with diameters in the range of 60–105 nm and primarily consist of Se(0), proteins and lipids. Moreover, Bio-SeNPs exhibit great potential for the removal of anionic dyes at pH 5 and cationic dyes at pH 10. The adsorption kinetics of dyes on the Bio-SeNPs can be characterized by the pseudo-second-order model. Isotherm test results revealed that the adsorption isotherms of dyes on the Bio-SeNPs fit the Langmuir equations well. The dye removal efficiencies increased with increasing temperature in the range of 303–323 K. Adsorption thermodynamics analysis showed that Δ*H*^θ^ were in the range of 1–9 kJ/mol, and Δ*G*^θ^ values were negative. This indicated that these adsorption processes were spontaneous and were primarily physical reactions. The adsorption capacities of Bio-SeNPs toward congo red, safranine T and methylene blue were 1577.7, 1911.0 and 1792.2 mg/g, respectively. The dye-removal ability is better than that of traditional biomass materials. In addition, the reusability of the Bio-SeNPs was successful after 5 cycles. 

The genetic strategy for constructing such genetically modified strains is easy to follow; since *Escherichia coli* S17-1 is a commercial strain, the *csrF* gene sequence can be found in NCBI GenBank database (Accession number, AAY72_00755) and *Alishewanella* sp. WH16-1 is available in the China Center for Type Culture Collection (=CCTCC M201507). Thus, following our procedures [19], researchers can construct other *E coli* strains with similar functions for deeper investigation or broad applications. 

## Figures and Tables

**Table 1:** Thermodynamic parameters for dye adsorption onto Bio-SeNPs.

Dyes	Δ*H*^θ^ (kJ /mol)	Δ*S*^θ^ (kJ /mol K)	Δ*G*^θ^ (kJ/mol)		
			303 K	313 K	323 K
Congo red	1.4546	0.0755	−21.43	−22.1849	−22.9398
Safranine T	6.2332	0.0727	−15.8049	−16.5318	−17.2588
Methylene blue	8.5850	0.1153	−26.3652	−27.5181	−28.6710

**Table 2:** Comparison of adsorption capacity of anionic dyes by some bio-based adsorbents.

Adsorbent	Dye	*q_e_* (mg/g)	Reference
CNC-PVAm	Congo red	1469.7	[26]
Magnetic biomass activated carbon	Congo red	369.7	[44]
Rice straw based carbon	Congo red	531.4	[46]
	Methylene blue	527.6	
Teak wood bark	Methylene blue	914.6	[4]
Corncob Activated Carbon	Safranin T	1428.8	[47]
Carboxylated cellulose derivative	Safranin T	1429.7	[48]
Bio-SeNPs	Congo red	1577.7	This work
	Methylene blue	1792.2	
	Safranin T	1911.0	

## Supplementary Materials

The following are available online at http://www.mdpi.com/2079-4991/8/4/234/s1, Table S1: Parameter values of the kinetics models fitting to the experimental results for adsorption, Table S2: Langmuir, Freundlich and Temkin adsorption isotherm constant, correlation coefficient and *q_m_*.

## Abbreviations

Bio-SeNPs	biogenic Se(0)-nanoparticles
DLS	dynamic light scattering
TEM	transmission electronic microscopy
HAADF	high-angle annular dark field
SEM	scanning electron microscopy
EDS	energy-dispersive X-ray spectrographs
FT-IR	Fourier transform infrared spectroscopy
XPS	X-ray photoelectron spectroscopy
