# Supplementary material for: Adsorption Removal of Multiple Dyes Using Biogenic Selenium Nanoparticles from an Escherichia coli Strain Overexpressed Selenite Reductase CsrF
Source: Nanomaterials (Basel). 2018 Apr 12;8(4):234. doi: 10.3390/nano8040234 (PMC5923564; doi:10.3390/nano8040234)
Supplement: Supplementary file 1 [file nanomaterials-08-00234-s001.pdf]

Adsorption removal of multiple dyes using biogenic selenium nanoparticles from an *Escherichia coli* strain overexpressed selenite reductase CsrF

Xian Xia, Zijie Zhou, Shijuan Wu, Dan Wang, Shixue Zheng, Gejiao Wang*

State Key Laboratory of Agricultural Microbiology, College of Life Sciences and Technology, Huazhong Agricultural University, Wuhan, 430070, P. R. China

* To whom correspondence should be addressed.

Gejiao Wang.

Tel: +86 27 87281261.

Fax: +86 27 87280670.

E-mail: gejiao@mail.hzau.edu.cn

Table S1 Parameter values of the kinetics models fitting to the experimental results for adsorption.

	Dyes	Congo red			Safranine T			Methylene blue		
	C_0 (mg/L)	50	100	200	50	100	200	50	100	200
First order	R^2	0.9896	0.9883	0.9918	0.9692	0.9546	0.955	0.9989	0.9998	0.9996
	k_1	0.3443	0.2252	0.088	0.21486	0.1778	0.1228	0.4819	0.8016	0.7282
	q_e (mg/g)	122.3874	225.6526	404.5659	51.3773	132.5186	208.8018	119.8046	240.1069	479.0459
Second order	R^2	0.999	0.9991	0.9984	0.9962	0.9901	0.9584	0.9996	0.9999	0.9999
	k_2	0.0062	0.0017	0.0002	0.0065	0.0019	0.0007	0.0146	0.0339	0.0115
	q_e (mg/g)	128.2158	243.0198	480.3693	55.8186	146.2223	237.6316	122.4501	241.4132	482.8749
Intraparticle diffusion	R^2	0.7697	0.7608	0.9047	0.9205	0.9338	0.6838	0.5588	0.74327	0.8294
	k_3	3.6569	11.1039	42.7581	2.9222	9.0313	17.61	1.5704	0.8428	2.5099
	c	100.6528	156.5418	110.1183	33.46762	76.2189	91.6559	110.4062	235.2992	464.7706

Table S2 Langmuir, Freundlich and Temkin adsorption isotherm constant, correlation coefficient and q_m.

	Dyes	Congo red			Safranine T			Methylene blue		
	Temperature (K)	303	313	323	303	313	323	303	313	323
Langmuir	R^2	0.9743	0.9518	0.9722	0.9909	0.9788	0.9836	0.9835	0.9437	0.9705
	K_L (L/mg)	0.0071	0.00715	0.00736	0.00139	0.0015	0.00162	0.0937	0.10323	0.11572
	q_m (mg/g)	1072.1224	1337.0217	1577.7388	1369.6945	1671.7905	1911.0299	1363.3269	1587.2356	1792.1568
Freundlich	R^2	0.9258	0.9459	0.9247	0.9652	0.9303	0.9361	0.8356	0.725	0.7731
	K_F (mg/g)	97.5378	117.6155	132.6498	16.5630	20.8831	26.1396	546.9868	655.5122	720.3662
	n	2.9285	2.8706	2.7975	1.7924	1.7914	1.8182	6.7682	6.9686	6.6489
Temkin	R^2	0.9632	0.9479	0.9552	0.9716	0.9745	0.9829	0.9105	0.8012	0.8551
	A	-565.7184	-592.6483	-777.6358	-1130.5648	-1500.5638	-1702.2231	333.0497	414.798	460.1529
	B	223.9262	260.9136	322.5697	277.3888	361.0862	415.6558	167.6103	191.8999	221.9619
